# An entropy-reducing data representation approach for bioinformatic data

**DOI:** 10.1093/database/bay029

**Published:** 2018-04-05

**Authors:** Alan F McCulloch, Ruy Jauregui, Paul H Maclean, Rachael L Ashby, Roger A Moraga, Aurelie Laugraud, Rudiger Brauning, Ken G Dodds, John C McEwan

**Affiliations:** 1 AgResearch, Invermay Agricultural Centre, Mosgiel, New Zealand; 2 AgResearch, Grasslands Research Centre, Palmerston North, New Zealand; 3 AgResearch, Lincoln Research Centre, Christchurch, New Zealand

## Abstract

Non-semantic approaches to bioinformatic data analysis have potential relevance where semantic resources such as annotated finished reference genomes are lacking, such as in the analysis and utilisation of growing amounts of sequence data from non-model organisms, often associated with sequence-based agricultural, aqua-cultural and environmental sampling studies and commercial services. Even where rich semantic resources are available, semantic approaches to problems such as contrasting and comparing reference assemblies, and utilising multiple references in parallel to avoid reference bias, are costly and difficult to fully automate. We introduce and discuss a non-semantic data representation approach intended mainly for bioinformatic data called *non-semantic labelling*. Non-semantic labelling involves tensorially combining multiple kinds of model-based entropy-reducing data representation, with multiple representation models, so as to map both data and models into dual metric representation spaces, with goals of both reducing the statistical complexity of the data, and highlighting latent structure via machine learning and statistical analyses conducted within the dual representation spaces. As part of the framework, we introduce a novel algebraic abstraction of data representation mappings, and present four proof-of-concept examples of its application, to problems such as comparing and contrasting sequence assemblies, utilisation of multiple references for annotation and development of quality control diagnostics in a variety of high-throughput sequencing contexts.

**Database URL**: https://github.com/AgResearch/data_prism

## Introduction

To find structure in high-entropy datasets we need to throw away information via entropy-reducing data representations that simplify the data while preserving structural features of interest. *Sequence annotation* involving labelling DNA or protein sequence observations with matches [e.g. top hits from BLAST (1)] in a reference database is a good example of such a representation: the information content (entropy) of the set of labels is much less than that of the original data, but the biological meaning of the original data is preserved assuming each original sequence is represented by a ‘functionally equivalent’ (in some sense) matched hit in the reference dataset. This is a *semantic* data representation technique, in that the original observations are represented as linguistic objects such as reference sequence names or blocks of text in the reduced dataset.

By contrast, calculating the mean and standard deviation of a numerical sample, an everyday entropy-reduction technique applied to datasets, is a *non-semantic* entropy-reduction, in that each original observation is represented by an unlabelled point in a 2D metric space. As Stigler notes (2) ‘The taking of a mean of any sort is a rather radical step in an analysis. In doing this, the statistician is discarding information in the data; the individuality of each observation is lost…’. In this paper we outline and apply an entropy-reducing data representation approach intended mainly for bioinformatic data that we term *non-semantic labelling*, in which the semantic individuality of data values such as sequences and taxonomy names is lost, and the data is labelled by points in a high dimensional vector or tensor (i.e. vector-space product) metric space.

## Methods

### Algebraic abstraction of entropy-reducing data representation mappings

We use a simple algebraic formulation to describe and manipulate entropy-reducing data representation mappings, allowing us to combine and obtain new representations which would be difficult to access using purely verbal reasoning and descriptions. We interpret and formulate below some standard elementary summary statistics as entropy-reducing data representation mappings to illustrate how this abstraction works (without meaning to suggest that it would be particularly productive to adopt this notation in the standard cases used for illustration). Consider a dataset consisting of N elements $x_{i}$, i ranging from 1 to N which we represent using entropy-reducing data representation mapping operators M, S, R, etc. (as defined below); some representation mappings such as taking ranks are model-dependent (ranking depends on an ordering model), in which case we will index the operator symbol by model, for example $R_{j}$ refers to a representation operator that yields the rank of a data element according to ordering model j. In these next examples the raw dataset elements are numbers, but in general could be strings (such as DNA sequences) or composites such as arrays:

**Table:** 

The mean-value representation mapping operator M maps each dataset element $x^{i}\;$to a constant value $\overset{-}{x}$, the mean of the entire collection of dataset values $x^{1}\;$to $x^{N}$. Here ‘taking an average’ is thought of as an entropy-reducing data representation mapping that represents each data element by a point in a (1D) metric space, with all data elements in a collection represented by the same singular point.	$M(x^{i})\;\to \;\overset{-}{x}$
The standard deviation operator S represents each dataset element $x^{i}\;$by the standard deviation of the entire collection of dataset values $x^{1}\;$to $x^{N}$	$S(x^{i})\;\to \;s$

Many entropy-reducing data representations are *injective* rather than aggregative—i.e. each data element is represented as a distinct point in the representation space:

**Table:** 

The ranking operator $R_{j}$ represents each dataset element $x^{i}\;$as the rank of that data element in the dataset, according to some ordering model $o_{j}$. Taking ranks is an entropy-reducing data representation, representing each data element as a point in a 1D space, but now the mapping is injective in that each element is represented by a different (depending on how ties are handled) point in the space.	$R_{j}\left(x^{i}\right)\to \;r_{j}^{i}$
Most entropy-reducing data representations are *partially* aggregative:	
The binning operator $B_{j}$ represents each dataset element $x^{i}\;$by the centre of a bin according to some binning model $b_{j}$ :	$B_{j}\left(x^{i}\right)\to \;c_{j}^{i}$
The empirical frequency operator $F_{j}$ represents each dataset element $x^{i}\;$by a count of how many elements fall into the parent bin of $x^{i}\;\;a$ccording to some binning model $b_{j}$:	$F_{j}\left(x^{i}\right)\to \;f_{j}^{i}$
The model-fitting operator G represents each dataset element by a fitted value according to some general model of the data.	$G(x^{i})\;\to \;{\hat{x}}^{i}$
We define a data representation transformation $I_{j}\;$ under which each data element $x^{i}\;$is represented by the self-information $h_{j}^{i}\;$of that element, relative to some probability model $m_{j}$ of the data, with $h_{j}^{i}=-\ln p_{j}\left(x^{i}\right)$ [$p_{j}$ the (often empirical) probability of $x^{i}\;$according to $m_{j}$]	$I_{j}\left(x^{i}\right)\to \;h_{j}^{i}$

The above entropy-reducing representation operators yield *scalar* representations: scalar representations can be combined to construct *vector* representations which represent data elements as vectors in a metric space of more than one dimension. For example:

**Table:** 

The entropy-reducing representation [M, S] represents each data element as a singular point $\left(\overset{-}{x},\;s\right)$ in a 2D space, corresponding to the mean and standard deviation of the collection. Various kinds of metric (usually based on the *t* distribution) can be constructed to assign a statistical distance between points in this space, and hence between the data collections they represent.	$\left[M,\;S\right]\left(x^{i}\right)\;\to (\overset{-}{x},\;s)$
The entropy-reducing representation $\left[B_{j},\;F_{j}\right]$ represents each data element as a point $\left(c_{j}^{i},\;f_{j}^{i}\right)$ in a 2D space, corresponding to the bin centre and membership count of the parent bin of $x^{i}$ according to a binning model $b_{j}$. The space corresponding to a particular binning model is usually visualised as a histogram.	$\left[B_{j},\;F_{j}\right]\left(x^{i}\right)\to (c_{j}^{i},\;f_{j}^{i})$

Scalar-valued entropy-reducing representations such as ranking, binning or self-information ($R_{j}$, $B_{j}$ & $F_{j}$ and $I_{j}$ above) are parameterised respectively by an ordering, binning or probability model so that vector-valued representations may also be constructed by indexing over multiple models. For example, vector-valued representations constructed by indexing over M probability or ordering models of the data could be expressed as:

$$\left[I_{1},I_{2},\ldots I_{M}\right]\left(x^{i}\right)\to (h_{1}^{i},h_{2}^{i},\ldots h_{M}^{i})$$

$$\left[R_{1},R_{2},{\ldots R}_{M}\right]\left(x^{i}\right)\to (r_{1}^{i},r_{2}^{i},\ldots r_{M}^{i})$$

Furthermore we can generalise in both of these ways—i.e. use both multiple *kinds* of entropy reducing representation, and multiple *models* associated with each representation. For example a ‘flat’ vector valued representation made by combining R and I and also indexing across M models could be expressed as:

$$\left[R_{1},R_{2},{\ldots R}_{M},\ldots I_{1},I_{2},\ldots I_{M}\right]\left(x^{i}\right)\to (r_{1}^{i},r_{2}^{i},\ldots r_{M}^{i},h_{1}^{i},h_{2}^{i},\ldots h_{M}^{i})$$

[Noble (3) refers to this way of combining different representations of the input data as ‘early integration’]. We have found it more useful to construct higher order representations by taking a formal tensor product of the model space with the space of representation operators. This yields a nested rather than flat data representation:

$$\left[\left[m_{1},m_{2},\ldots m_{M}\right]⊗\left[R,I\right]\right]\left(x^{i}\right)\to (\left(r_{1}^{i},h_{1}^{i}\right),\left(r_{2}^{i},h_{2}^{i}\right),\ldots \left(r_{M}^{i},h_{M}^{i}\right))$$

Vector-valued representations are then just special cases of this abstraction, for example

$$\left[\left[m_{1},m_{2},\ldots m_{M}\right]⊗\left[R\right]\right]\left(x^{i}\right)\to \left(r_{1}^{i},r_{2}^{i},\ldots r_{M}^{i}\right)$$

$$\left[\left[m_{1},m_{2},\ldots m_{M}\right]⊗\left[I\right]\right]\left(x^{i}\right)\to \left(h_{1}^{i},h_{2}^{i},\ldots h_{M}^{i}\right)$$

### Duality between data and representation models

There is an interesting and useful duality between data and models. Consider for example the vector representation:

$$\left[\left[m_{1},m_{2},\ldots m_{M}\right]⊗\left[I\right]\right]\left(x^{i}\right)\to (h_{1}^{i},h_{2}^{i},\ldots h_{M}^{i})$$

This characterises each data element $x^{i}$ by our ‘surprise’ at seeing it, as measured by its self-information according to a panel of M different probability models of the data (with low probability hence high self-information implying greater ‘surprise’). The dual expression:

$$\left[\left[x^{1},x^{2},\ldots x^{N}\right]⊗\left[I\right]](m_{j})\to (h_{j}^{1},h_{j}^{2},\ldots h_{j}^{N}\right)$$

exchanges the role of data and model: it characterises model $m_{j}$ by how surprising it is to see each of the data items according to that model. We refer to the representation $s^{i}=(h_{1}^{i},h_{2}^{i},\ldots h_{M}^{i})$ as the *spectrum* of data element $x^{i}$ relative to the panel of probability models, and the dual representation $s_{j}=(h_{j}^{1},h_{j}^{2},\ldots h_{j}^{N})$ as the *co-spectrum* of model $m_{j}$ relative to the dataset. For this particular representation operator, which is information based, we would refer to *information* spectra and co-spectra; for a panel of ranking operators we would refer to *ranking* spectra and co-spectra, etc. In these examples the spectra and co-spectra are vectors, but for higher order representations such as $\left[R,I\right]$ above they may be, for example matrices. As we will see in the examples below, sometimes we are mainly interested in the structure of the spectra, sometimes in the co-spectra, and sometimes both. This duality increases the flexibility of our data representation abstraction.

### Metric structure for data and models

Vector-valued entropy-reducing data representations endow distances between pairs of data elements (dually, pairs of models) via finding a suitable metric on the vector space of corresponding spectra (dually, co-spectra). This is provided at a high level of abstraction by a suitable inner product between pairs of spectra or co-spectra, whether vectors, matrices or higher order tensors. We illustrate the development of a number of inner-product based metrics for both vector and matrix spectra and co-spectra in the application examples below.

## Application examples

**Example 1:** Alignment of sequences against multiple references

In this example we aligned a dataset of 203 333 cattle expressed sequence tags (ESTs) (AgResearch, Genesis and Primary Industry Victoria Bovine EST Project 2006, unpublished data) to a panel of 10 sheep and cattle genomes and transcriptomes using BLAST, in order to

1. investigate the possibility of non-semantic annotation of these sequences, using alignments against multiple reference genome and transcriptome assemblies to embed the sequence collection in a metric representation space in which distances between sequence representations are related to biological distance; and
2. investigate the possibility of using the dual embedding of the panel of genomes and transcriptomes to investigate and visualise the relationships between them, via a suitable metric on the dual representation space.

The transcriptome and genome references are described in Table 1.

**Table 1.** The panel of assemblies used to label the EST dataset

Assembly name	Reference type	Species	Description
BovineVelvetSE	Transcriptome	Cattle	Assembly of Single End Illumina NGS Reads
cs39	Transcriptome	Sheep	Assembly of sheep EST sequences
cs34	Transcriptome	Cattle	Assembly of cattle EST sequences
dfciBt	Transcriptome	Cattle	DFCI Bos taurus gene indices
dfciOa	Transcriptome	Sheep	DFCI Ovis aries gene indices
Bgisheep	Genome	Sheep	BGI sheep genome assembly
Btau42	Genome	Cattle	Baylor College Cattle assembly 4.2
Btau461	Genome	Cattle	Baylor College Cattle assembly 4.6.1
Umd2	Genome	Cattle	University of Maryland Cattle assembly version 2
Umd3	Genome	Cattle	University of Maryland Cattle assembly version 3

In this analysis the EST collection is aligned against each assembly in turn, yielding a self-information measure for each EST relative to each assembly as follows: an EST that aligns at a locus in one of the assemblies at which many other sequences also align, is assigned a low self-information relative to that reference, whereas a sequence that aligns at a locus where no other sequences in the collection align, is assigned a high self-information relative to that reference.

Specifically, the self-information $h_{j}^{i}$ of each EST $x^{i}$ relative to reference $m_{j}$ is calculated as follows: each $m_{j}$ provides an empirical probability model of the sequence collection, with the empirical probability $p_{j}\left(x^{i}\right)$ associated with sequence $x^{i}$ taken as the alignment depth in a window centred on the alignment position of $x^{i}$ in $m_{j}$ divided by the total number of sequences that were aligned, and $h_{j}^{i}=-log(p_{j}\left(x^{i}\right))$.

A vector-valued entropy-reducing representation of the EST dataset is then obtained by representing each EST sequence $x^{i}$ by an information spectrum $s^{i}$ consisting of the respective self-information of that EST relative to each of the references. This representation is expressed using our abstraction as:

$$\left[\left[m_{1},m_{2},\ldots m_{10}\right]⊗\left[I\right]\right]\left(x^{i}\right)\to \left(h_{1}^{i},h_{2}^{i},.h_{10}^{i}\right)=s^{i}$$

Dually each reference is represented by an information co-spectrum $s_{j}$ relative to the EST dataset:

$$[\left[x^{1},x^{2},\ldots x^{203333}\right]⊗\left[I\right]](m_{j})\to \left(h_{j}^{1},h_{j}^{2},\ldots h_{j}^{203333}\right)=s_{j}$$

The information spectrum for each EST sequence provides a *non-semantic label* for it based on how ‘surprised’ we are to see the sequence, according to the alignment of the sequence collection against each of the reference assemblies. Dually, the information co-spectrum for each reference assembly provides a dual non-semantic label for it based on the pattern of self-information of the EST collection relative to that assembly. (We refer to these as kinds of *label* rather than as kinds of *data feature*, because they are constructed using a collection of external labelling models, rather than being intrinsic to the data; also the collection of models can be arbitrarily large, such that we could if useful *uniquely* label each data element.)

We developed an inner-product-based metric structure for the collection of ESTs based on their spectra, and for the panel of reference assemblies based on their co-spectra, as described in detail in the Supplementary Material.

**Figure 1.**
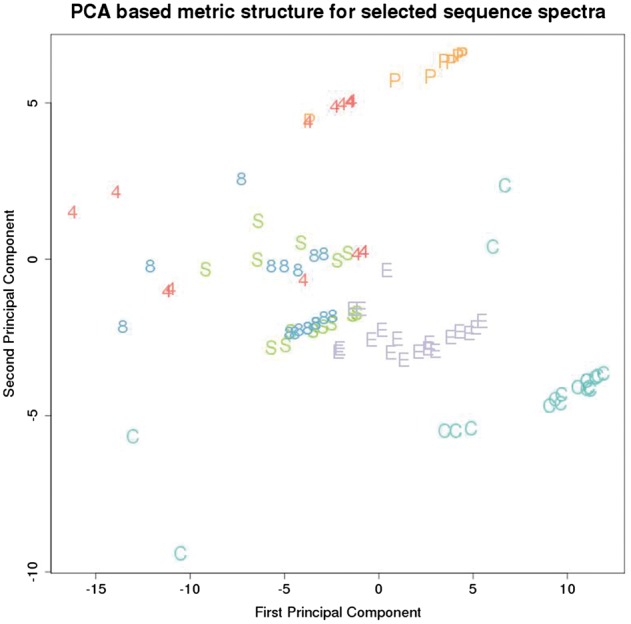
Metric structure on a selection of spectra for each of 120 EST sequences from the dataset. Each point in this plot represents an EST and is labelled by a letter corresponding to the EST gene, as listed in Table 2.

Figure 1 depicts the EST metric structure for a selection of EST’s representing the genes from Table 2.

**Table 2.** Gene names and single letter symbols plotted

Gene	Symbol
Prolactin (PRL)	P
Selenoprotein (SEP15)	S
Eukaryotic Translation Elongation Factor (EEF1A1)	E
Keratin (KRT4)	4
Keratin (KRT8)	8
Alpha-S1-casein (CSN1S1)	C

These genes were chosen among those that were well represented in the dataset, across a range of different biological functions. The distance between each pair of EST’s in Figure 1 corresponds directly to the distance assigned by the metric.

This metric segregates the different genes reasonably well, although there is considerable overlap between KRT4, KRT8 and SEP15. Interestingly a close biological association between keratinocytes and selenoproteins has been noted (4).

**Figure 2.**
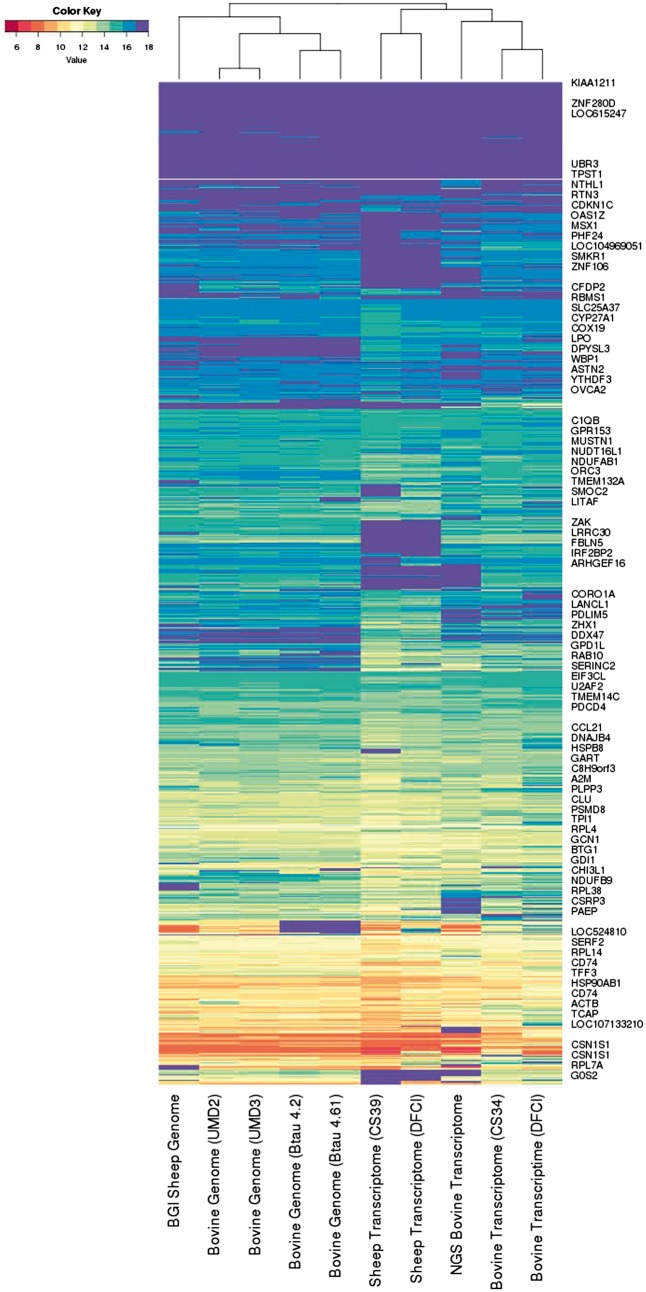
The information spectra and co-spectra for the EST dataset. Each row depicts the spectrum of an EST, and each column depicts the co-spectrum of a reference.

As described in the Supplementary Material we also developed an inner-product-based metric on the reference assembly *co-spectra*, which mathematically involves clustering the EST *spectra*. The dendrogram in Figure 2 provides a visualisation of this metric based on 300 distinct EST clusters, mostly ranging in size from 30 to 2500 sequences (with only two large sequence clusters outside this range, of sizes 7567 and 13 714). This choice of metric captures salient technical and biological relationships between the assemblies:

- The five transcriptomes on the right are distinguished from the five genomes on the left.
- Within the genomes, the sheep reference on the left segregates from the four cattle genomes, and the UMD and Baylor assemblies segregate together respectively.
- Within the transcriptomes the two sheep transcriptomes on the left segregate from the three cattle transcriptomes on the right, and the short-read-based cattle transcriptome segregates from the two Sanger-based transcriptomes on the far right.

We found that metrics based on <100 or >600 clusters captured some but not all of these features, so that it appears a roughly 300 dimensional co-spectrum sub-space is required to capture all of these features. This metric will have the effect of giving roughly equal weight to each gene or gene family, whereas a Euclidean metric would give greater weight to highly expressed genes.

In Figure 2 each row depicts the full information spectrum

$$\left[\left[m_{1},m_{2},\ldots m_{10}\right]⊗\left[I\right]\right]\left(x^{i}\right)\to \left(h_{1}^{i},h_{2}^{i},.h_{10}^{i}\right)=s^{i}$$

for each sequence $x^{i}$.

**Figure 3.**
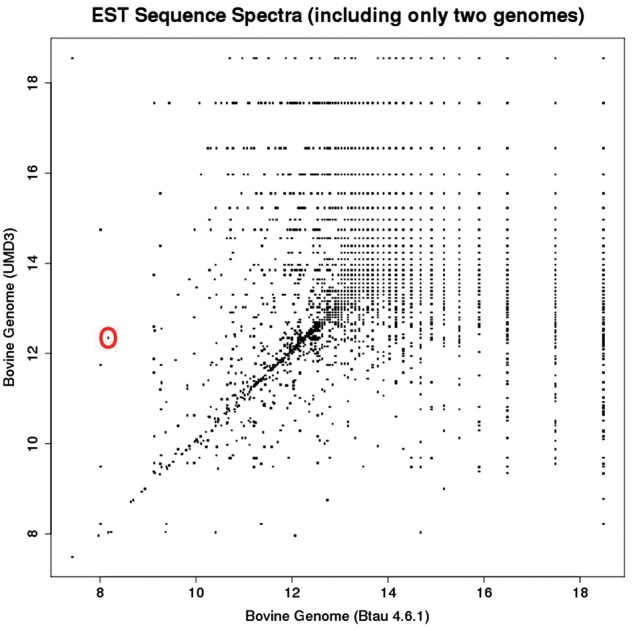
2D sub-spectra for all EST sequences in the dataset. Each point in this plot corresponds to one EST sub-spectrum, but often corresponds to multiple ESTs (which have the same sub-spectrum). Whereas the full EST spectrum has 10 elements, the sub-spectra depicted here have 2 elements.

By including only two assemblies in the representation operator, we can plot the spectrum of each sequence as a point in a 2D visualisation. Figure 3 depicts the information spectra

$$\left[\left[m_{Btau4.6.1},m_{umd3}\right]⊗\left[I\right]\right]\left(x^{i}\right)\to \left(h_{Btau4.6.1}^{i},h_{umd3}^{i}\right).$$

While Figure 2 provides a “birds-eye-view” of the relationships between references via the metric structure endowed by their co-spectra, Figure 3 enables drilling down to specific regions of potential difference represented by off-diagonal points on a plot of 2D EST sub-spectra. For example the off-diagonal point that is circled discloses [via the UCSC Liftover tool (5)] a 1.5 MB rearrangement in UMD3 relative to Btau4.6.1, with a segment of this length at chr5: 9 500 000 in Btau4.6.1 moved to chr5: 104 000 000 in the UMD3 assemblies. Rearrangements such as these change the probabilities that sequences align, thus changing the information spectrum for a sequence (as well as influencing the dual co-spectra and metric distance between assemblies).

**Figure 4.**
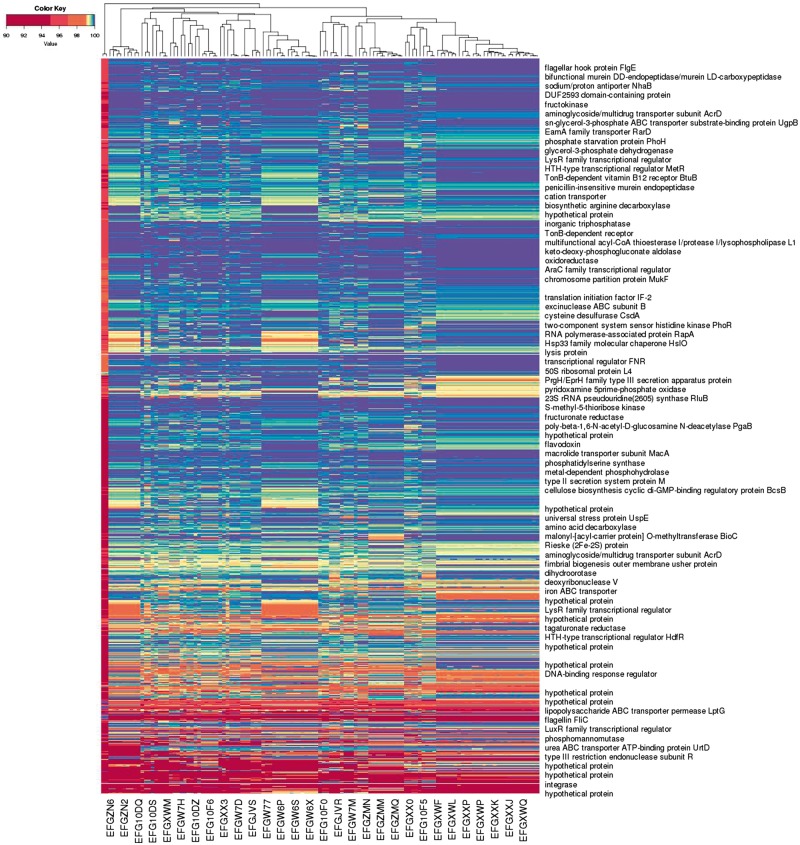
Alignment-based spectra of 1 055 875 oligo probes, and co-spectra of the 122 unfinished bacterial strain genome assemblies that the probes were aligned against. Each row depicts the average spectrum of a cluster of probes, with row labels based on annotation of a representative probe from the cluster; each column depicts the co-spectrum of a bacterial assembly. (Only some rows and columns are labelled.)

Figure 4 illustrates an application of this approach to a larger number of assemblies. The representation form $[\left[x^{1},x^{2},\ldots x^{1055875}\right]⊗\left[PctID\right]](m_{j})\to \left(b_{j}^{1},b_{j}^{2},\ldots b_{j}^{1055875}\right)=s_{j}$ labels each of 122 unfinished bacterial strain genome assemblies $m_{j}$ with a co-spectrum $s_{j}$ in which $b_{j}^{i}$ is the percentage identity of the best BLAST hit of oligo $x^{i}$ to assembly $m_{j}$, as described in detail in the Supplementary Material. Each assembly consists of a set of unordered un-oriented contigs which makes direct alignment-based (i.e. semantic) comparisons difficult to interpret. The non-semantic approach was useful in giving us a quick visualisation of the broad relationships between the bacterial strains, with segregation of the assemblies matching expectations based on the characteristics of the samples.

**Example 2:** Summarising blast results obtained as part of sequencing centre quality control

An important quality control step in any sequencing lab processing taxonomically diverse sequencing projects involves blasting a random sample of short reads from each distinct output batch against a reference database in order to look for contamination and processing errors such as barcode swaps and labelling errors. The choice of reference database is a trade-off between sensitivity, specificity and speed. For non-model organisms the full NCBI nt nucleotide database (6) is sometimes used as it is fast and fairly sensitive, but has poor specificity, with many hits to gene homologs and paralogs, leading to a fairly high-entropy dataset consisting of counts of hits to a very large number of taxa, which can be difficult to interpret. Interpretation is usually semantically based, involving ignoring all but the two or three highest-frequency taxa in the tabulation of hits and subjectively assessing whether the hit taxa adequately match the sample description. As well as discarding less frequent but potentially informative taxa hits this also discards contextual across-samples information (‘how does this sample compare with others generated by the facility’) which may be relevant to a diagnosis of possible contamination or systematic error.

To develop an intuitive visualisation of blast results that utilises *all* taxa hits and also contextual information we adopted a non-semantic approach based on considering the tabulation of *all* taxonomy name hit counts as a high dimensional non-semantic label of a sample, without interpreting the taxonomy names. This also allows us to use contextual information by clustering samples so that we can check how the latest sample clusters with previous samples from the centre.

As described in detail in the Supplementary Material each batch is represented by an information co-spectrum $s_{j}$ relative to the collection of all taxonomy names observed in a blast search of a random sample of sequences from each batch:

$$[\left[x^{1},x^{2},\ldots x^{T}\right]⊗\left[I\right]](m_{j})\to \left(h_{j}^{1},h_{j}^{2},\ldots h_{j}^{T}\right)=s_{j}$$

where T is the number of distinct taxonomy names hit across all batches, currently approaching 4000 for a typical series of sequencing lanes in a facility handling a diverse range of species.

**Figure 5.**
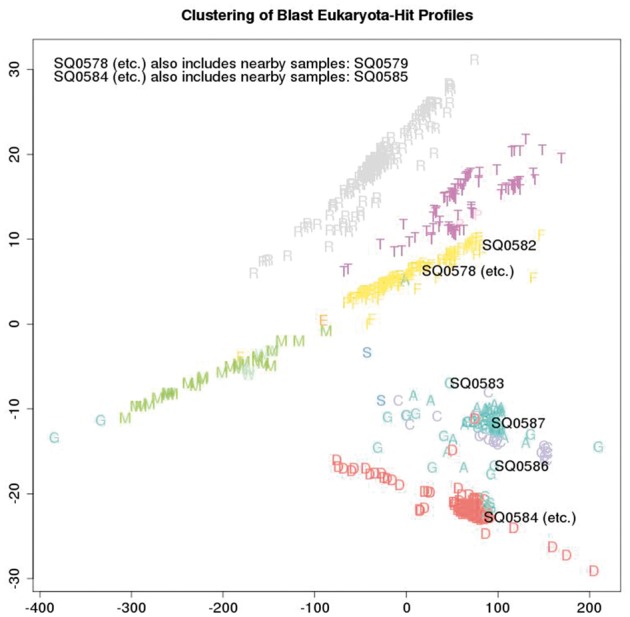
A total of 659 distinct samples from a cumulative series of 109 Illumina Hiseq flow-cells are plotted and labelled by colour and a single letter to indicate the nominal species that was sampled. All cumulative samples are plotted but the sample ID numbers (SQnnnn) of just the eight latest samples under review are overlaid, to visualise how the biological content of the latest samples compares with previous samples.

Figure 5 depicts the metric structure of the cumulative collection of sequencing batch co-spectra as at a certain date, with the nominal sample species of each batch coded with a single letter as in Table 3. (Usually this is the actual species, unless a batch is comprised of two or three species multiplexed, in which case the letter corresponds to one of the species.) The main commercial species handled by the lab can be clearly seen (ryegrass—the cluster of grey R; salmon—the cluster of yellow F; white clover—the cluster of purple T; Mussel—the cluster of green M; Deer—the cluster of red D; sheep, cattle and goat—the cluster of A, C and G). Outliers are usually due to multiplexing of multiple species in a single lane. The metric is again inner-product based.

**Table 3.** Sample details and single letter species symbols plotted

Species	Symbol	Sample ID (current run)	Description
sheep	A	SQ0578	Atlantic Salmon (all)
salmon	F	SQ0579	Atlantic Salmon (all)
cattle	C	SQ0582	Chinook Salmon (all)
deer	D	SQ0583	Atlantic Salmon (125), Goat (167)
seal	S	SQ0584	Deer (all)
goat	G	SQ0585	Deer (all)
mussel	M	SQ0586	Deer (69), Goat (315)
pea	P	SQ0587	Goat (all)
ryegrass	R		
clover	T		
weevil	W		
fungus	E		

This application relates to a lab that processes mainly eukaryotic samples and we are interested in quickly picking up events such as miss-labelling, cross-contamination between eukaryotic samples, and bacterial contamination. As part of the quality control process associated with each run of eight samples, this plot is generated: all cumulative samples are included in the analysis but the sample ID numbers of just the eight latest samples under review are overlaid. A metric on the space of sample co-spectra yields a distance matrix consisting of distances between each pair of sequencing batches and this is embedded in a 2D visualisation using multi-dimensional scaling. This visualisation of the q/c blast results provides immediate indication whether or not there is evidence of systematic error due to events such as mislabelling, or significant between-sample contamination from different species, by providing an immediate visual answer to the question “how do these samples compare taxonomically with previous samples from the same species?”. Thus rather than using just the most frequent blast-top-hit taxa in a batch to annotate it, we use *all* of the blast-top-hit taxa counts to non-semantically label each batch (with its co-spectrum as above), with metric structure on the space of labels then providing a sensitive internal consistency check of current versus previous batches.

**Example 3:** k-mer analysis of a de-novo sequence assembly project

We characterised sequencing data from three fungal strains as part of quality control and diagnostic work associated with de-novo fungal genome assemblies using these data, by summarising the DNA 6-mer frequencies in a 0.001 random sample of each of the 16 sequencing data-files involved. The samples and sequencing are described in Table 4.

**Table 4.** Fungal genus and sequencing protocol

Sample name	Sequencing method	Genus
C7N0GANXX-1804-01-4-1	Illumina paired-end	*Epichloë*
C7N0GANXX-1804-01-7-1	Illumina mate-pairs	
C7N0GANXX-1804-02-4-1	Illumina paired-end	*Lecanicillium*
C7N0GANXX-1804-02-7-1	Illumina mate-pairs	
C7N0GANXX-1804-03-4-1	Illumina paired-end	*Acremonium*
C7N0GANXX-1804-03-7-1	Illumina mate-pairs	
C7N0GANXX-1804-04-4-1	Illumina paired-end	*Acremonium*
C7N0GANXX-1804-04-7-1	Illumina mate-pairs	

As described in detail in the Supplementary Material each 6-mer $x^{i}$ is represented by an information spectrum $s^{i}$consisting of the respective self-information of that 6-mer relative to each of the files of sequencing data:

$$\left[\left[m_{1},m_{2},\ldots m_{16}\right]⊗\left[I\right]\right]\left(x^{i}\right)\to \left(h_{1}^{i},h_{2}^{i},.h_{16}^{i}\right)=s^{i}$$

Dually, each sequencing file is represented by an information co-spectrum $s_{j}$ relative to the collection of all possible 6-mers:

$$[\left[x^{1},x^{2},\ldots x^{4096}\right]⊗\left[I\right]](m_{j})\to \left(h_{j}^{1},h_{j}^{2},\ldots h_{j}^{4096}\right)=s_{j}$$

**Figure 6.**
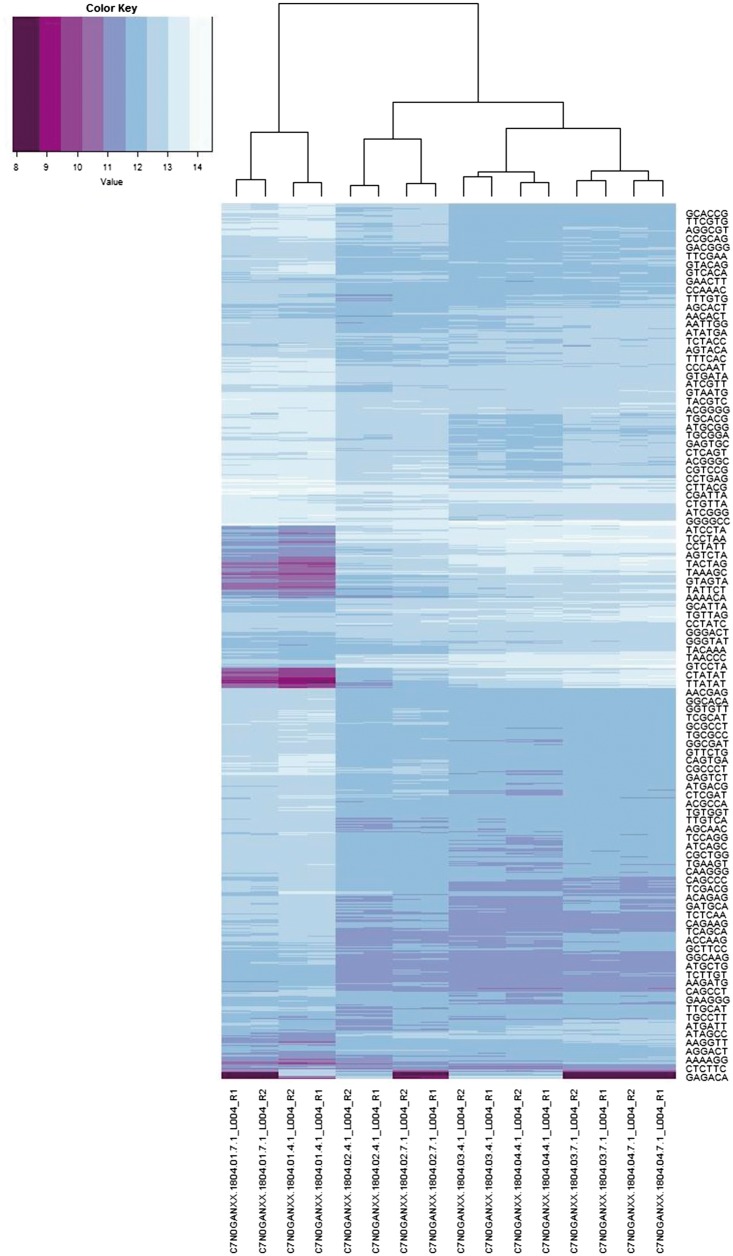
6-mer spectra (rows) and sample sequencing file co-spectra (columns). The dendrogram and row clustering are based on a standard Euclidean metric (only every 40th 6-mer is labelled).

In this application we are interested in both the spectra and co-spectra, which are depicted in Figure 6. The main structure noticeable visually in the sample co-spectra is the segregation of the three species Epichloë (left-most four columns), Lecanicillium (next four columns) and Acremonium (remaining eight columns). A notable feature of the 6-mer spectra is a band of low self-information (i.e. relatively more frequent) 6-mers (indicated by darker row colouring) in the Epichloë samples. We confirmed that this is related to the abundance of short repeats in Epichloë (7) (∼1% of its genome) by extracting sequence fragments exhibiting the 140 most enriched 6-mers and searching these back against a database of repeat consensus sequences from (7) and obtaining significant matches. Another notable feature of the 6-mer spectra is the narrow alternating pattern of high and low self-information at the bottom of the plot. Unlike the other band of low self-information 6-mers, this pattern does not appear to be related to a biological feature, because it segregates by sequencing method rather than by sample. We confirmed that the 33 6-mers from this part of the spectrum could be assembled into a complete adapter contaminant.

To further explore the structure of this dataset, we developed a tensorial representation form involving both ranking and self-information operators, yielding a matrix-valued rather than vector-valued co-spectrum for each sample:

$$[\left[x^{1},x^{2},\ldots x^{4096}\right]⊗\left[R,I\right]](m_{j})\to (\left(r_{j}^{1},h_{j}^{1}\right),\left(r_{j}^{2},h_{j}^{2}\right),\ldots \left(r_{j}^{4096},h_{j}^{4096}\right))=s_{j}$$

**Figure 7.**
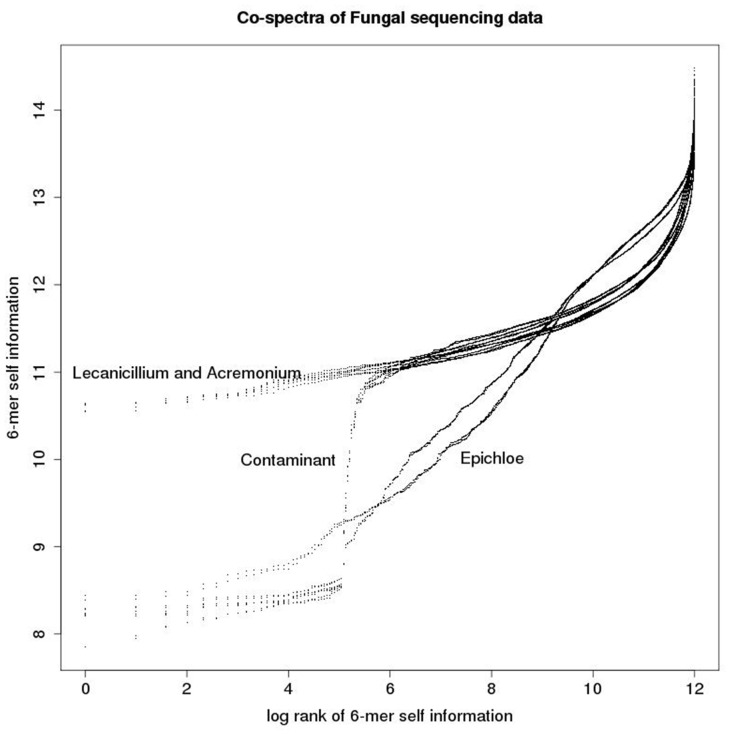
Each co-spectrum matrix consists of a list of ordered pairs and can be thought of as defining a functional relationship between rank and self-information as depicted in this plot.

These co-spectra are depicted in Figure 7, in which each co-spectrum matrix defines a functional relationship between log rank and self-information. We refer to these as *zipfian* plots, because they are closely related to the plots of log frequency versus log rank often used to investigate power-law relationships (the difference being that we rescale frequencies as probabilities and change sign after taking the log to obtain a self-information measure—hence our plots have positive rather than the typical negative slope of zipf power-law plots). The main structure noticeable visually is the predominantly linear relationship between log rank and self-information (apart from a steep tail in the right hand part of the plots)—this is due to the commonly observed power law relationship between the rank and frequency of DNA words in genomes. The slope of the linear sections segregates by species, with the Epichloë samples having a steeper slope than the Acremonium and Lecanicillium samples. As noted above the Epichloë genome is known to be relatively repeat-rich, and the steeper slope is due to the presence of a number of low-information 6-mers associated with these repeats. This feature suggests that the Acremonium and Lecanicillium genomes are expected to be less repeat rich than the Epichloë genomes, and similarly repeat-rich to each other. There is also a prominent turning point in the plot, a non-biological feature as it segregates by sequencing method rather than species. It is due to the presence of adapter contamination, as was also disclosed by the heat-map plot.

The advantage of the matrix-valued representation form is its ability to clearly visually highlight a number of different biological and technical features of the data via first and second derivatives of the co-spectrum functions, such as relative repeat content (slope, i.e. first derivative), and adapter contamination (turning points, i.e. second derivative).

## Application Example 4: k-mer analysis of tags from a genotyping-by-sequencing project

Genotyping by sequencing (GBS) (8) is a molecular technique which itself implements an (in-vitro) entropy reducing representation of genomic sequence, using restriction enzyme-based sampling to avoid relatively uninformative repetitive regions of genomes and efficiently target more informative lower copy regions. After enzyme digestion sample fragments are barcoded so that multiple samples can be sequenced on a single sequencing-machine lane, thus yielding a powerful and cost-effective assay of genomic variation. While the final product consists of allele counts by sample and locus, an important intermediate product after sequencing and de-multiplexing is typically a set of per-sample frequency distributions summarising the numbers of all distinct medium length (64 bp in this example) DNA tags.

While high-throughput technologies such as GBS are powerful, they depend critically on the integrity of long and complex chains of meta data and data processing, so that it is important to deploy a range of complementary processing metrics as part of quality controlling the process as a whole. As part of such a wider suite of quality control metrics, we individually characterise and contrast these tag distributions by summarising DNA 6-mer frequencies in each and obtaining a co-spectrum matrix representation of each individual sample:

$$[\left[x^{1},x^{2},\ldots x^{4096}\right]⊗\left[R,I\right]](m_{j})\to (\left(r_{j}^{1},h_{j}^{1}\right),\left(r_{j}^{2},h_{j}^{2}\right),\ldots \left(r_{j}^{4096},h_{j}^{4096}\right))=s_{j}$$

**Figure 8.**
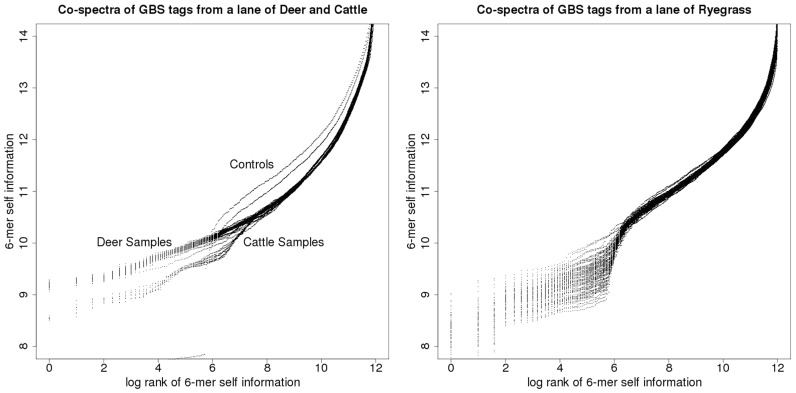
GBS tag-derived co-spectrum matrices from two sequencing lanes, the lane on the left nominally cattle, the lane on the right ryegrass.

Figure 8 depicts two examples of such co-spectrum spaces: the left-hand plot of Figure 8 depicts co-spectrum matrices for 192 GBS samples, recorded as being from cattle; the right-hand plot of Figure 8 depicts co-spectrum matrices for 96 GBS samples, recorded as being from ryegrass. As above, each co-spectrum matrix defines a functional relationship between log rank and self-information, so that respectively 192 and 96 distinct functions appear on each of these plots.

In the cattle set there were two negative-control samples and these can be visually distinguished (the two outlying curves in the left-hand plot, which lie above the cluster of biological samples in the right-hand part of the plot, and step down to the two flat sections in the left-hand part of the plot); whereas in the ryegrass plot the two negative controls can’t be distinguished, suggesting a technical difference in plate preparation methods between the two species.

The relationship between log rank and self-information is again linear in the main part of the cattle plot, without any sharp turning point, while the ryegrass plot exhibits a central turning point which steps down to a flatter part of the curve indicating a small set of co-abundant enriched (i.e. low self-information) 6-mers, probably due to biased and variable sampling of one or more ryegrass repeats.

The parts of these plots at the right margins which turn fairly sharply upward represent rare 6-mers (i.e. high self-information): we have noted that turning points in these kinds of plots correspond to non-biological sampling artefacts—for example in Figure 7 the turning point corresponds to an adapter contamination (and we have noted in other work that amplicon-based sequence data yields plots of this type that curve prominently along their entire length). We think it is likely that the high self-information 6-mers represented by the steeply curved right-hand margin of these plots are due to non-biological sampling artefacts such as infrequent sequencing errors.

There are two broad clusters of spectra visually apparent in the cattle plot, with slightly different slopes. This suggested that the samples from this lane were not taxonomically homogenous, since we find empirically that sequence data from different species have different slopes when characterised in this way, and vice-versa, taxonomically homogenous GBS lanes exhibit only a single cluster of this kind of spectrum. It turned out that indeed a small number of deer samples (corresponding to the lower cluster) had been included in this lane of predominantly cattle samples.

**Figure 9.**
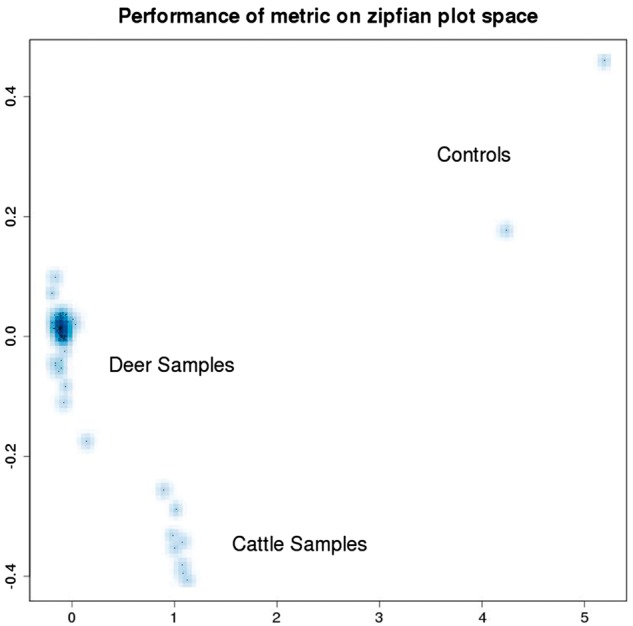
Distances between pairs of co-spectra depicted in the left-hand plot (cattle and deer) of Figure 8 have been calculated using a metric, and the resulting distance matrix embedded in a 2D visualisation using multi-dimensional scaling.

In order to enable computational clustering of these matrix co-spectra to complement and confirm the clusters that are apparent from visual inspection, we developed an inner-product-based metric that can be used to assign distances between pairs of co-spectra: this involves summing the squared differences in self-information, inversely weighted by rank—details of this formula and its derivation are given in the Supplementary Material. Figure 9 illustrates the metric structure thus obtained, on the space of cattle and deer sample co-spectra depicted in the left-hand plot of Figure 8 that is yielded by our metric.

## Discussion

In their chapter *Large Scale Data Representations* [chapter 5 in *Frontiers in Massive Data Analysis* (9)], the *National Research Council Committeeonthe Analysis of Massive Data* (NRC Committee) describe data representation as *the choice of a mathematical structure with which to model the data or, relatedly, to the implementation of that structure* and go on to distinguish three basic kinds, five broad goals and six challenges and future directions of large-scale data representation. This discussion focuses mainly on mapping our work onto the NRC Committee’s taxonomies of data representation kinds, goals and challenges in order to clarify the provenance, goals and contribution of the non-semantic labelling approach we describe above.

The three kinds of data representation given by (9) are: *Basic data structures* such as hash tables, indexes etc.; *More abstract, but basic, mathematical structures*, such as sets, vectors, matrices, graphs and metric spaces; *Derived mathematical structures*, such as clusters, linear projections, data samples. In this paper we present an approach which yields dual tensorial (i.e. vector, matrix or higher) representations of both data and models, with corresponding dual metric structures: this is an example of the NRC committee’s second category, ‘*More abstract, but basic, mathematical structures*’. As alluded to in their opening definition of ‘data representation’, there is a continuum between the initial data representation stage of data analysis and subsequent analysis stages that utilise those representations (e.g. via machine learning and/or statistical modelling). Thus for example, while our work focuses on data representation, rather than machine learning or statistical modelling, we find ourselves using statistical modelling and machine learning tools such as k-means clustering and principal components analysis internally, to mathematically induce a metric on the data representation space. (See for example the Supplementary Material for Example 1, where we explain how this works mathematically, with clustering of the EST spectra corresponding to projection of the reference assembly co-spectra, hence yielding a metric on the co-spectra.)

According to (9), there are typically five broad goals of data representation: Reducing Computation; Reducing Storage and/or Communication; Reducing Statistical Complexity and Discovering the Structure in the Data; Exploratory Data Analysis and Data Interpretation; Sampling and Large-Scale Data Representation. Our data representations are tensorial combinations, of scalar entropy-reducing representation mapping operators such as $I_{j}$ (which represents a data element by its self-information according to a probability model of the data), or of more technical representation operators such as representing a sequence by the percentage identity in a BLAST alignment with another sequence from a database (as used in the representation presented in Figure 4). Each scalar-valued representation reduces the statistical complexity of the data, without eliciting much structure, however by taking a tensorial combination of a number of complementary representation operators, we obtain dual high dimensional entropy-reducing representations which are able to reveal structure: we refer to these as spectra and co-spectra, and generically as ‘non-semantic labels’. They support exploratory data analysis and interpretation, and reflect the main goals of our work which are (in terms of the NRC taxonomy) Reducing Statistical Complexity and Discovering the Structure in the Data, and Exploratory Data Analysis and Data Interpretation. Although not the main focus of our work to date, our data representations, which live in a metric space, also support metric query-by-example of large-scale databases, via calculating distances between the data representation of an exemplar query, and a database of representations. The result of such a query is a ‘ball’ of query ‘matches’ that are within a given radius of the query: this aspect falls under the goals of Reducing Computation, and Sampling and Large-Scale Data Representation.

Six challenges associated with data representation at large scales that are highlighted by the NRC Committee are: How to Extend Existing Methods to Massive Data Systems; Heavy-Tailed and High-Variance Data; Primitives: Develop a Middleware; Manipulation and Integration of Heterogeneous Data; Understanding the Relative Strengths of Data-Oblivious and Data-Aware Methods; Combining Algorithmic and Statistical Perspectives. In their discussion of the Challenge of Primitives, the committee calls for ‘…[identification of] a set of primitive algorithmic tools that (1) provide a framework to express concisely a broad scope of computation; (2) allow programming at the appropriate level of abstraction…’. We have outlined an algebraic abstraction of data representation in which data representations can be expressed concisely at a high level of abstraction via algebraic combination of lower order representation operators: thus for example in Example 1, on the face of it Figure 2 appears to be doing something quite different to Figure 1, however the abstraction clarifies that these are simply algebraic duals of one another, with Figure 1 visualising the representation $\left[\left[m_{1},m_{2},\ldots m_{10}\right]⊗\left[I\right]\right]\left(x^{i}\right)\to \left(h_{1}^{i},h_{2}^{i},.h_{10}^{i}\right)=s^{i},$ and Figure 2 visualising the dual representation $[\left[x^{1},x^{2},\ldots x^{203333}\right]⊗\left[I\right]](m_{j})\to \left(h_{j}^{1},h_{j}^{2},\ldots h_{j}^{203333}\right)=s_{j}$. Similarly Figure 3 appears to be a different representation again, but is in fact just a lower dimensional version of Figure 1, as made clear by its algebraic form $\left[\left[m_{Btau4.6.1},m_{umd3}\right]⊗\left[I\right]\right]\left(x^{i}\right)\to \left(h_{Btau4.6.1}^{i},h_{umd3}^{i}\right)$. In Example 3 we are able to concisely interpret a set of zipf plots of log k-mer frequency versus log rank, as being matrix-valued data representations $[\left[x^{1},x^{2},\ldots x^{4096}\right]⊗\left[R,I\right]](m_{j})\to (\left(r_{j}^{1},h_{j}^{1}\right),\left(r_{j}^{2},h_{j}^{2}\right),\ldots \left(r_{j}^{4096},h_{j}^{4096}\right))=s_{j}$. We suggest that this kind of concise high-level abstraction could contribute to programing data representations at a higher level of abstraction as the NRC Committee calls for.

As noted by the NRC Committee in their discussion of the challenge of *Manipulation and Integration of Heterogeneous Data*, and also by other authors such as Alpaydin [in his discussion in Chapter 17 in (10)], a common data representation approach of ‘putting everything into a feature vector’, has a number of drawbacks. We showed in Example 3 how a higher order *tensorial* (matrix valued in this case) data representation was able to reveal structure not apparent from a vector representation, and went on to show how to develop an inner-product-based metric on this kind of representation. While the suggestion of using tensor rather than vector-based data representation is not new [see, e.g. Cai et al. (11)], our work contributes usefully in several ways: first by presenting a straightforward practical application of a higher order (than vector) tensorial data representation; second as we have discussed by introducing an expressive algebraic abstraction of higher order data representations; third by showing an example of developing a metric on a space of higher order representations, at a high level of abstraction (which we did by looking for an inner product on the space of matrix-valued representations, interpreted as functions).

We offer an entropy-reduction perspective on another of the NRC challenges—*Combing Algorithmic and Statistical Perspectives*. An interesting and important potential of non-semantic data representation not utilised explicitly in this paper (nor very often in bioinformatics, but standard practice in statistical modelling and analysis) is *quantitation* of latent structure via analysis of residuals—i.e. of differences between the original data and its representation: if residuals exhibit a maximum entropy probability distribution then we know we have ‘thrown away as much information as possible’—so that it will not be possible to find any other representation which will extract more structure from the data. For example the normal distribution has maximum entropy among all distributions with a specified variance, so that normally distributed residuals indicate that the entropy-reducing data representation used (e.g. this could be a model that was fitted) has extracted as much structure from the data as possible, by throwing away as much information as possible—i.e. the representation is ‘optimal’ in that sense. By contrast assessing whether we have thrown away as much information as possible in a bioinformatic *semantic* entropy-reducing representation such as contig assembly (e.g. that we have not under-assembled) is complex and mainly qualitative.

While we cannot calculate residuals between the data originals and representations for the kinds of representation mappings applied in our test cases (such as mapping DNA sequence strings to numeric self-information vectors), we *can* make inferences from the distributional properties of the lower-entropy data representations—i.e. of the numeric spectra and co-spectra. For example if there is a good fit to these by a maximum entropy distribution such as the multi-variate normal, then since the information content of the original data themselves before entropy-reducing representation is by design higher than that of their data representations, it follows that the original data features must also have a maximum entropy distribution, and hence we can infer that little or no structure in those original data features is available. Conversely if, as in the cattle plot in Figure 8 of Example 4, the distribution of co-spectra representations clearly does not have a maximum entropy distribution (i.e. here it is bi-modal), then we can infer the existence of latent structure in the data—and indeed it turned out there was more than one species sequenced. Our inference that there was latent structure was really based on an implicit premise, that the data representations do not have a maximum-entropy distribution: in this case that was obvious (they have a bi-modal distribution), but in general it would be necessary to statistically test the truth of this premise. Our point here is that the non-semantic data representation approach we have outlined potentially supports quantitative rather than just qualitative judgements about the existence of latent structure in typical bioinformatic datasets consisting of sequence strings and other non-numeric data items.

Finally, an important data representation challenge not mentioned by the NRC Committee, relates to increasing the scope and utilisation of new computational paradigms such as quantum computing, which will require innovative data representations. The rapid progress in bioinformatics and computational biology in the last 30 or so years has been made possible by extraordinary advances in micro-processing power as described by Moore’s law, which observes that since around 1965 the number of transistors on a processor doubles every 2 years. However there is general agreement that the limits of this technology have been reached and that we will no longer be able to rely on Moore’s law to deliver compute platforms capable of solving problems currently out of reach: ‘In future the revolution will have to continue by other means’(12). Recent advances in quantum computer technology and algorithm development suggest these other means will include quantum computing (13), while quantum computing appears likely to be relevant both to specific hard bioinformatics problems such as protein fold prediction (14), and also to classes of algorithms relevant to bioinformatics such as optimisation and constraint satisfaction; graph problems such as determining connectivity, minimum spanning trees and shortest path; pattern matching; Markov chain-based searches and sampling (via quantum walks); solving linear equations; data fitting and various tasks in machine learning (15). By representing bioinformatic objects such as sequences, reference assemblies and sample data files in high dimensional linear spaces, with a rich metric structure, which are particularly suited to a quantum computing approach, a non-semantic data representation framework of the kind we have sketched could have a role to play in providing data representations suitable for quantum computing platforms of the near future.

## Summary and conclusions

We described and applied a data representation framework, *non-semantic labelling*, that embeds datasets in dual metric spaces, together with a novel algebraic abstraction of data representation which offers concise expression, manipulation, communication and implementation of data representation mappings. Using this framework we demonstrated as a proof-of-concept non-semantic sequence annotation using alignments against multiple reference genome and transcriptome assemblies to represent the sequence collection in a metric space in which distances between sequence representations are related to biological distance; and shown how the dual metric representation of genomes and transcriptomes highlights important technical and biological relationships between them, including for example specific structural differences such as rearrangements via investigating outlier sequence representations. This non-semantic approach is scalable to large numbers and various different classes of assemblies, including unfinished and un-annotated.

We showed how “zipfian” *k*-mer (*k* = 6 in our examples) self-information versus log rank plots, can be obtained abstractly as a formal tensor product of a 2D entropy-reducing data representation operator (ranking, binning) with a vector of representation models (in the form of sequence data-files), yielding a matrix-valued representation which clearly reveals data features not apparent from lower order scalar or vector representations. This suggests that algebraic abstraction of data representation could contribute to the development of other higher order data representations to aid visualisation and understanding of large and complex datasets.

We found that the first derivative (slope) of the zipfian function plots suggested by the framework tended to broadly segregate sequence data by species of origin, while the second derivative (turning points) helps identify technical and biological sequence data features such as adapter contamination and relatively highly abundant repeats. We hypothesise that turning points in this kind of plot are *always* non-biological in origin, due, for example, to untrimmed adapter fragments or biased molecular sampling and have found the presence of this feature to be a reliable and easy to obtain diagnostic indicator of these kinds of event. We note that de-novo assembly of co-abundant low-self-information 6-mers as disclosed by zipfian plots into complete contaminants and in some cases (e.g. associated with biased sampling of repeats) repeat units is straightforward and useful and as part of further work are investigating a non-semantic information-based sequence trimming and filtering approach based on sculpting the zipfian plot to remove turning points, which would not require any prior knowledge of contaminant sequence.

We have discussed how our approach is subsumed under taxonomies of data representation approaches, goals and challenges outlined in (9) by the NRC Committee, and drawn attention to its potential relevance to the emerging challenge of designing data representation algorithms to enable wider utilisation of the new computing paradigm of quantum computing.

## Supplementary data

Supplementary data are available at *Database* Online.

## Supplementary Material

Supplementary Data
