# Supplementary material for: An entropy-reducing data representation approach for bioinformatic data
Source: Database (Oxford). 2018 Apr 5;2018:bay029. doi: 10.1093/database/bay029 (PMC5887302; doi:10.1093/database/bay029)
Supplement: Supplementary Data [file bay029_supp.doc]

# An Entropy-Reducing Data Representation Approach for Bioinformatic Data

# Supplementary Material

# Metric structure for data and models

Vector valued data representations endow distances between data elements (dually, between models) via a suitable metric on the vector space of corresponding spectra (dually, co-spectra). Useful metric structure on these spaces is often provided by a suitable inner product
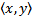
between pairs of spectra or co-spectra.


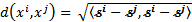
 (distance between a pair of data points)


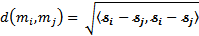
 (distance between a pair of models)

# Application Example 1: Metric Structure on the collection of ESTs

Inner products of pairs of sequence spectra provide a metric structure for the collection of sequences according to which a distance endowed between a pair of sequences
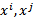
 is:


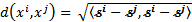


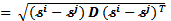


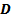
is a symmetric matrix.

To construct
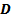
 in this example, we used Principal Components Analysis (PCA) to project the information spectrum of each sequence into a lower dimensional sub-space, and then took the standard Euclidean inner product (dot product) on the projected spectra. Projecting into a sub-space spanned by vectors
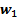
and
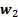
 we have


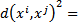


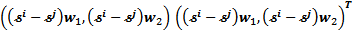


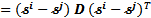


with


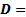

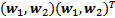


We used the first two eigenvectors of the covariance matrix of the information spectra to provide
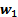
 ,
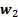
 (i.e. here we are interpreting PCA as yielding a non-Euclidean metric
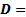

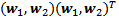
 on the space of information spectra, so that the distance between each point in the PCA plot is identical to the distance assigned by the metric).

# Application Example 1: Metric Structure on the panel of reference assemblies

Dually, inner products of pairs of co-spectra provide a metric structure for the set of reference assemblies:


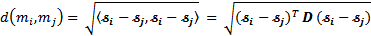


To obtain
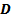
 we again used the approach of projecting the co-spectrum vectors into a sub-space, taking the Euclidean inner product of projected vectors. Since *projection* of co-spectra and *clustering* of spectra are duals of each other we can obtain a projection of the assembly co-spectra via clustering the sequence spectra. We used the k-means algorithm to cluster the sequence information spectra, with each cluster represented by the centre of the cluster. Thus:


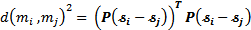


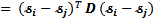


with


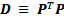


and
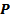
 a
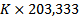
 weighted incidence matrix that clusters
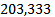
 sequence spectra into
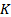
 clusters.
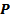
is implicitly determined by the dual k-means clustering of the spectra, and neither
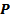
 nor
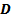
 are explicitly evaluated.

# Application Example 1: Scaling up to a larger number of unfinished assemblies, and using a different reduction operator.

As noted in the main text, the reduction form
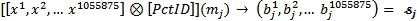
labels each of 122 unfinished bacterial strain genome assemblies
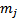
with a co-spectrum
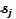
in which
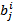
 is the percentage identity of the best BLAST hit of oligo
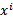
 to assembly
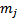
, with metric structure on the co-spectra obtained by clustering the spectra as above. Rather than using a self-information based reduction operator as was done with the EST dataset of Example 1, the reduction operator used here maps each sequence to its percentage identity with the best BLAST alignment to the reference. Details are as follows.

We extracted 1,055,875 (redundant) overlapping 1kb probes from the pooled sequence of 122 unfinished bacterial strain genome assemblies and searched these back against each of the assemblies using BLAST, recording the percentage identity of the best hit of an oligo to a contig in the assembly. In order to obtain a metric on the assemblies (i.e. co-spectra), the k-means algorithm was used as above to cluster the spectra, into 10,000 clusters. Note that in Figure 2, the clustering process yielded the metric structure (i.e. distance matrix, of distances between spectra) used to visualise the EST spectra, but the clusters themselves are not retained – it is the original EST spectra that are plotted; in Figure 4 however the clusters are retained and it is the centres of the clusters of spectra that are plotted. We produced Figure 4 this way for a technical reason: it is simpler to use the heatmap package to both generate the distance matrix and also produce the plot, rather than pre-calculate the distance matrix based on the clusters, and then pass this and the original un-clustered data to the heatmap function for plotting (i.e. as was done for Figure 2). Conversely, the clustering-based distance matrix was pre-calculated for Figure 2, but the original un-clustered data was plotted, because we wanted to emphasise that the role of the clustering is to obtain a data metric, rather than to smooth the presentation of the data. The simpler procedure used for Figure 4 is technically preferable in that the code is simpler and shorter which reduces the chances of error, and plotting the cluster centres rather than the original data reduces the noisiness of the plot and thus may assist interpretation.

# Application Example 2: Summarising Blast Results obtained as part of Sequencing Centre Quality Control

In this application example a 1 in 20,000 random sample of each cumulative batch of sequencing (usually a lane of Illumina sequencing) is adapter trimmed and then searched against the genbank nt database using blast, with options *-evalue 1.0e-10 -dust '20 64 1' -max_target_seqs 1 -outfmt ‘qseqid sseqid pident evalue staxids sscinames scomnames sskingdoms stitle'*, and only the top alignment is retained. The taxonomic output fields are concatenated to yield a taxonomic common name assignment for each query: these names are often not the same as the actual taxonomic common name for the sample, even in the absence of any contamination, because the nt database though comprehensive is incomplete, and also the query may be identically homologous to a database sequence with different taxonomy. However as described in the text, taken as a whole the collection of all the assigned common taxonomy names yields a spectral signature for each batch which is specific as to its taxonomic composition and can be used to taxonomically cluster the batches. This is done as follows. Each batch can be considered to provide an empirical probability model of the collection of blast-hit common taxonomy names; the empirical probability
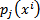
 associated with taxonomy name
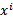
 by batch
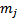
 is the number of sequences from that batch with top-hit to an nt accession annotated with taxonomy name
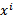
, divided by the total number of sequences that were blasted. As in the above example, this is used to calculate the self information of taxonomy name
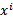
 according to
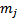
 ,
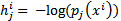


A vector valued representation of the collection of all taxonomy names that have been observed is provided by an operator which maps each taxonomy name
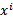
 to an information spectrum
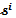
consisting of the respective self information of that taxonomy name relative to each of the batches:


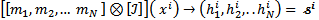


where
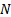
 is the cumulative number of batches from the sequencing centre (around 700 in our example).

Dually each batch is mapped to an information co-spectrum
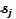
relative to the collection of taxonomy names:


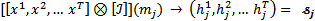


where
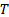
 is the number of distinct taxonomy names hit across all batches, currently approaching 4,000 for a typical series of sequencing lanes in a facility handling a diverse range of species.

The information spectrum of each taxonomy name is thus a vector (with length equal to the cumulative number of sequencing batches, currently around 700 in our example) and labels it, based on the pattern of self-information of that name across the batches; dually, the information co-spectrum of each batch is a vector (of length around 4,000 in our example) that labels it based on the pattern of self-information of taxonomy names within that batch. In this application we are interested in developing a measure of how taxonomically similar sequencing batches are to one another so that we are interested in the co-spectra. The metric on the space of co-spectra is again based on Euclidean distances between projected co-spectra, with the projection based on dual k-means clustering of the spectra (as described above in example 1).

# Application Example 3: k-mer analysis of a de-novo sequence assembly project

In this application each file of sequence data provides an empirical probability model of DNA 6-mers. The empirical probability
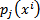
 associated with a 6-mer
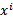
 by file
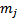
 is the count of
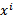
in
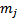
, divided by the total count of all the 6-mers in
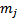
. As above this is used to calculate the self-information of 6-mer
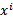
 according to
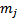
 ,
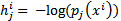


As described in the main text, a vector valued representation of the collection of all 6-mers’s that have been observed is provided by an operator which maps each 6-mer
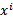
 to an information spectrum
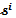
consisting of the respective self information of that 6-mer relative to each of the files of sequencing data:


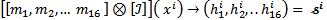


Dually each sequence file is mapped to an information co-spectrum
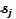
relative to the collection of all 4,096 DNA 6-mers


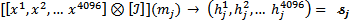


The information spectrum of each 6-mer is thus a vector (of length 16 in this dataset) and labels it, based on the pattern of self-information of that 6-mer across the data files; dually, the information co-spectrum of each file is a vector (of length 4,096) that labels it based on the pattern of self-information of 6-mers in sequences in that file. In this application we are interested in both the spectra and co-spectra, which are depicted in Figure 6 in the main text.

Figure 7 in the main text depicts the tensorial reduction form involving both ranking and self-information operators, yielding a matrix-valued rather than vector-valued co-spectrum for each sequencing file:


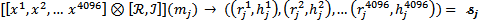


Here the rank
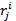
assigned to a 6-mer
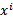
 by ranking operator
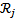
 is simply the rank of that 6-mer in an ordering of all 6-mers based on their self-information in the sequence file. A matrix valued spectrum for each 6-mer
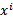
 could be constructed in a similar way, but this is not done here.

A note on the choice of 6-mers (as opposed for example to 5-mers): In the literature, both pentamers and hexamers have been used to construct feature vectors, for example as input to machine learners, to try to predict biological function. In that context it tends to be important to try to limit the dimensionality of the feature vector to avoid "curse of dimensionality" problems, and pentamers may be used partly for that reason, as the dimension of the vector is then at most 1024 as compared with 4096 for hexamers. (However even when pentamers are used in that context, 1024 tends to be too large and analysts will usually tend to use some kind of binning of different pentamer frequencies to reduce the length of the vector). In our context however we are only (to date) using the hexamer based data representation for unsupervised learning (i.e. clustering), and for this they give useful results. While pentamers would probably also give useful results here, we prefer hexamers because one of our analyses involves picking out clusters of co-abundant k-mers and assembling these so as to accomplish de-novo annotation of repeats and contaminants, and for this kind of assembly the longer hexamers are strongly preferred.

# Application Example 4: k-mer analysis of tags from a genotyping-by-sequencing project

To develop a metric on the co-spectrum matrix space we note that each co-spectrum matrix defines a functional relationship between log rank and self-information, so that inner products of pairs of these functions would be a reasonable basis for a metric. Thus we can assign a distance between co-spectrum matrices and hence between sequencing files
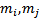
 as:


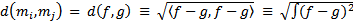


with the
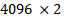
dimensional matrix co-spectrum of each file taken as defining the functional relationships


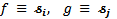


Thus


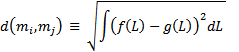


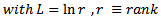


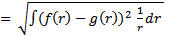


This argument suggests a metric for assigning distances between samples that is based on distances between their respective co-spectrum matrices as


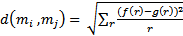
 (1)

This metric will have the effect of giving less weight to unusual observations (i.e. to rare 6-mers in this example) which seems reasonable. Potential modifications include modifying the weighting
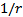
, or simply restricting the range of integration (i.e. zero-weighting some parts of the function). Alternatively a different kind of metric based on the first and second derivatives of the co-spectrum functions could be considered, as we found these to be related to biological and technical properties of the samples.

# Supporting Software

As part of this work we developed a utility *data_prism.py* to generate the spectra and co-spectra labels described in the paper. *data_prism.py* builds spectra from large input data-files (for example fasta, fastq, bam, vcf), containing mixed continuous and discrete multivariate data (the continuous variables are binned). Features include random sampling, multi-processing to improve throughput, caching distribution data so that spectra can be incrementally extended, and calculation of distance matrices on the spectra.

*data_prism.py* contains a small kernel of generic methods for binning, calculating self-information etc., with data parsing being handled by call-backs implemented in client code, so that the kernel has no dependency on specific data formats such as fasta, fastq, vcf, bam etc. The software is available at <https://github.com/AgResearch/data_prism>. This repository includes a number of examples of using *data_prism.py* with various different types of input such as tab-delimited text, fasta and fastq, bam files, vcf files.
